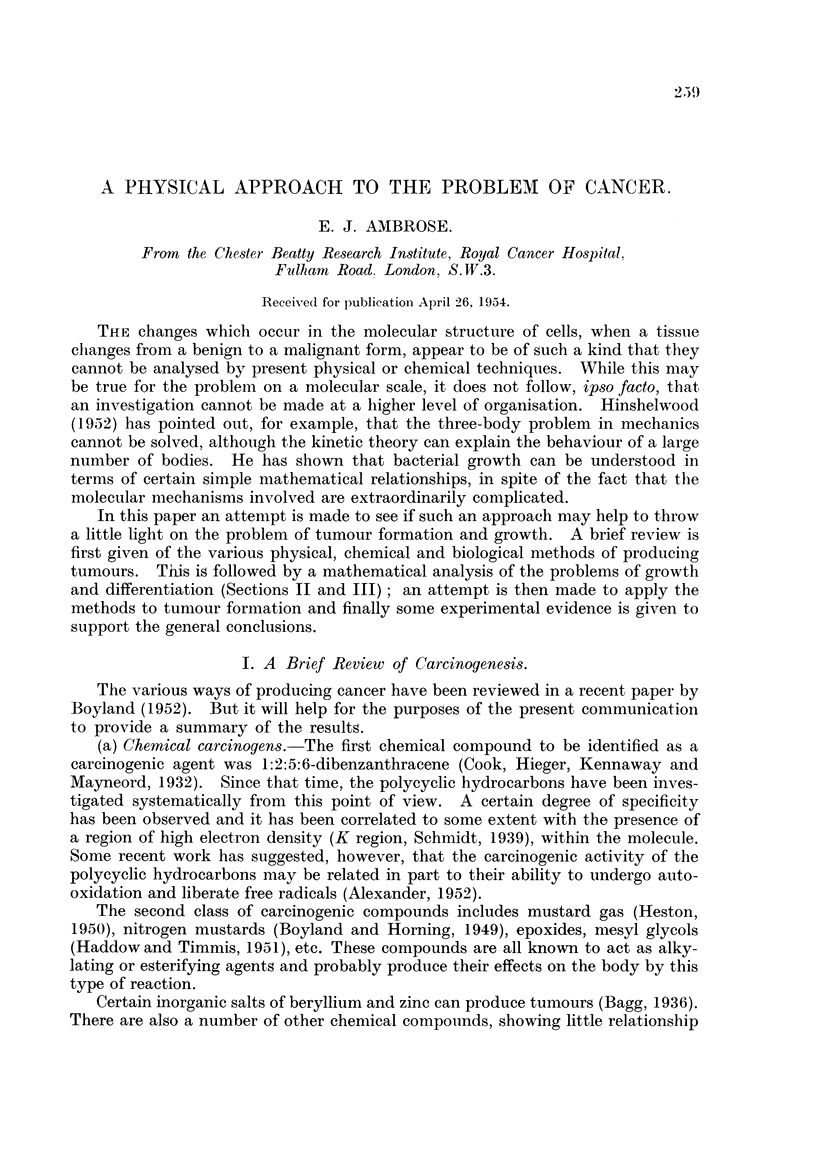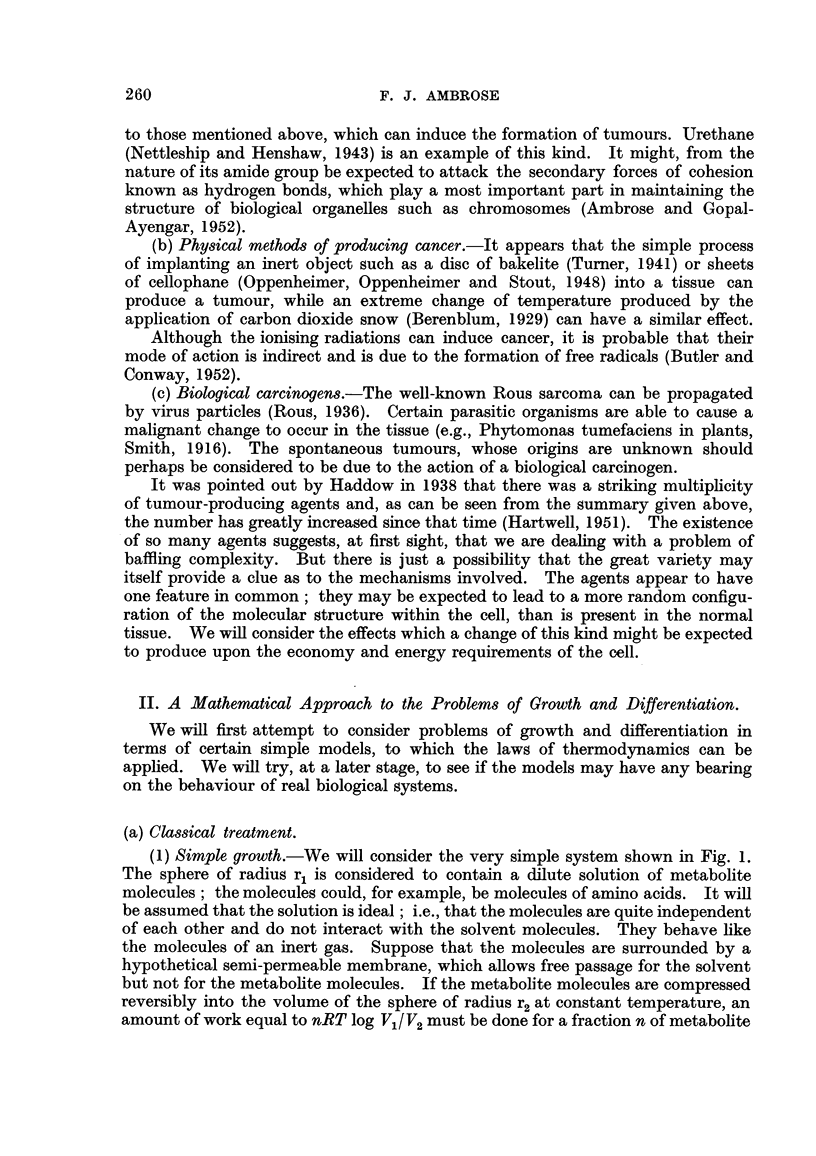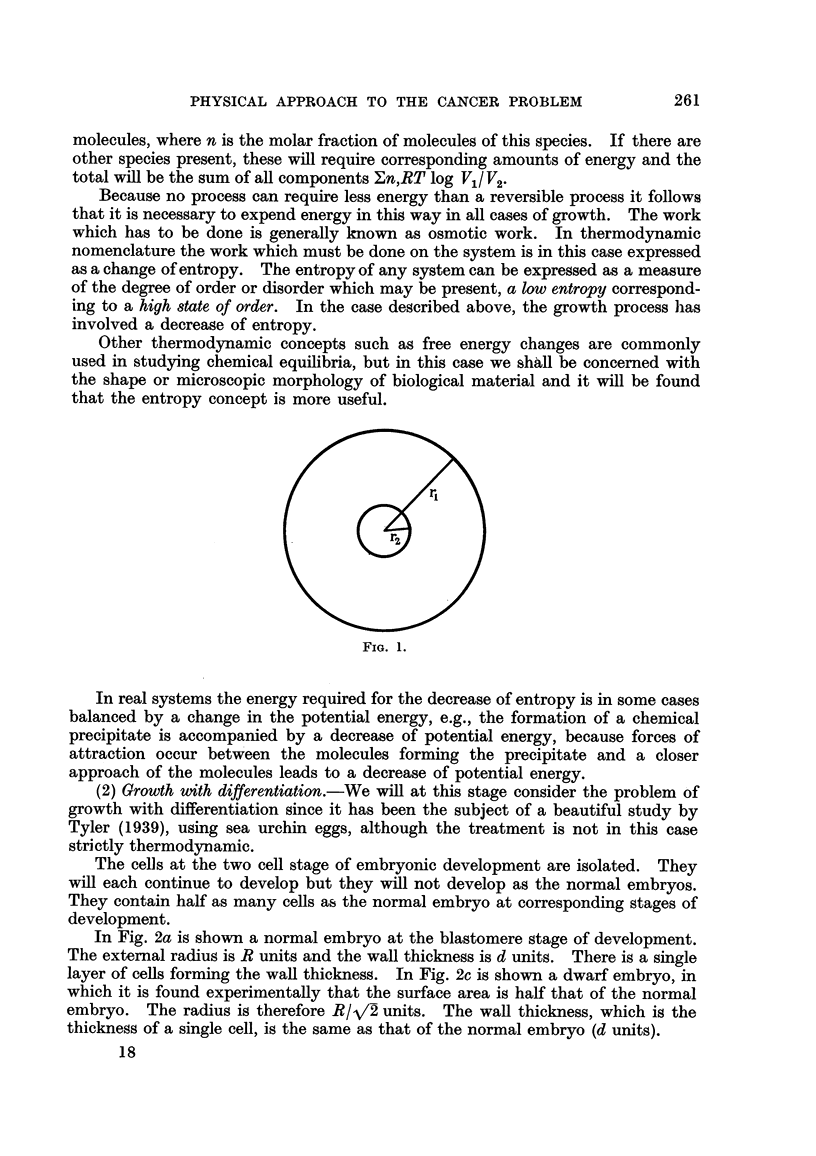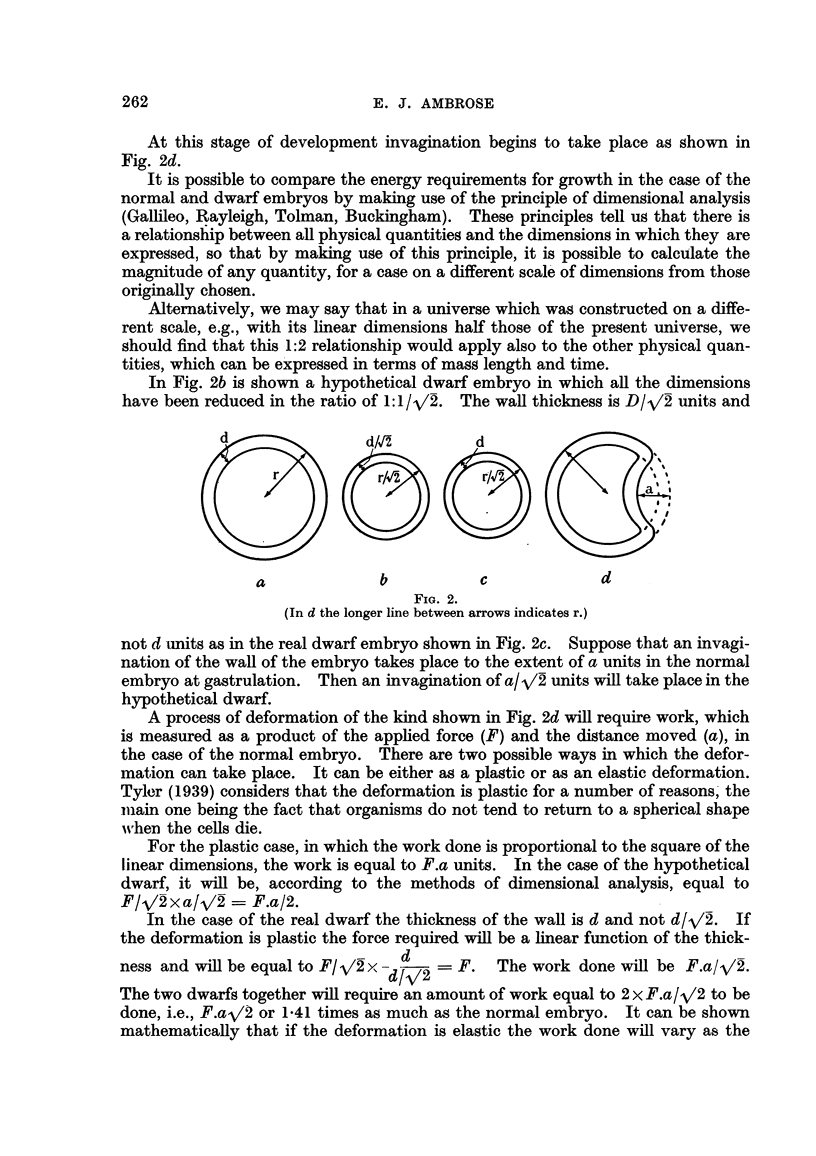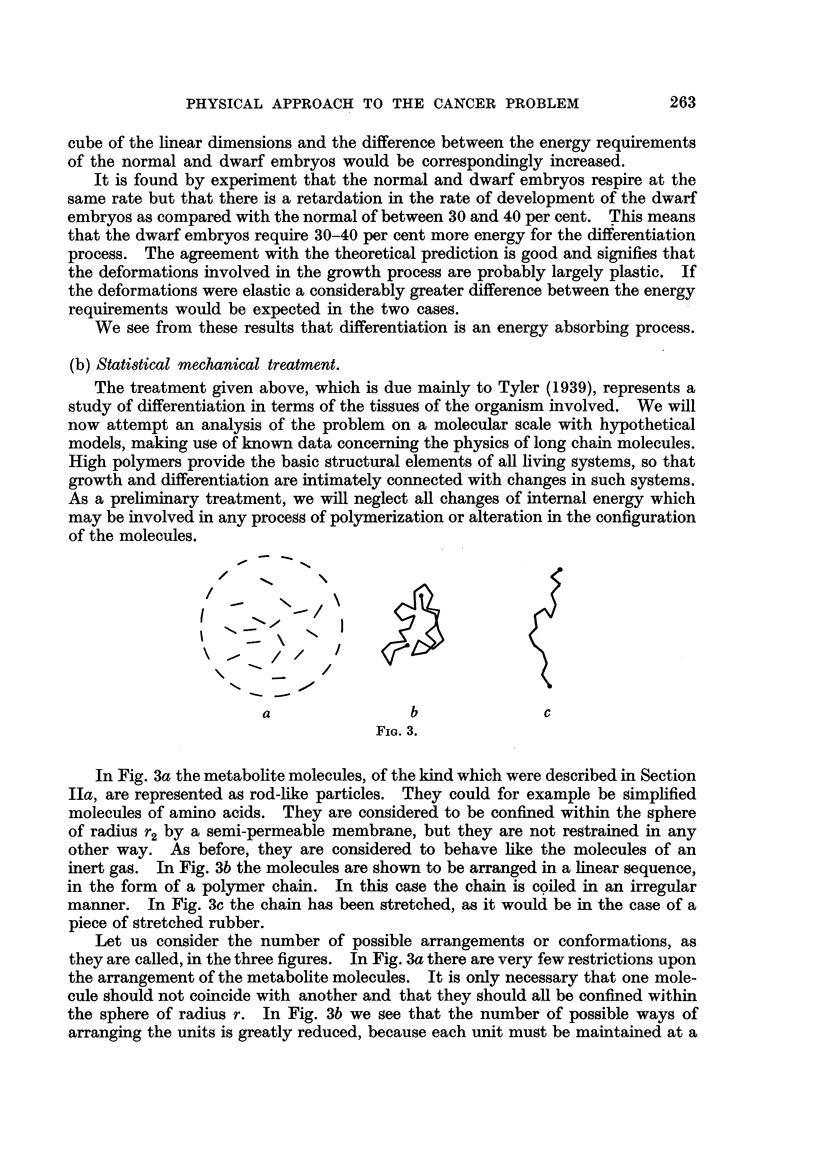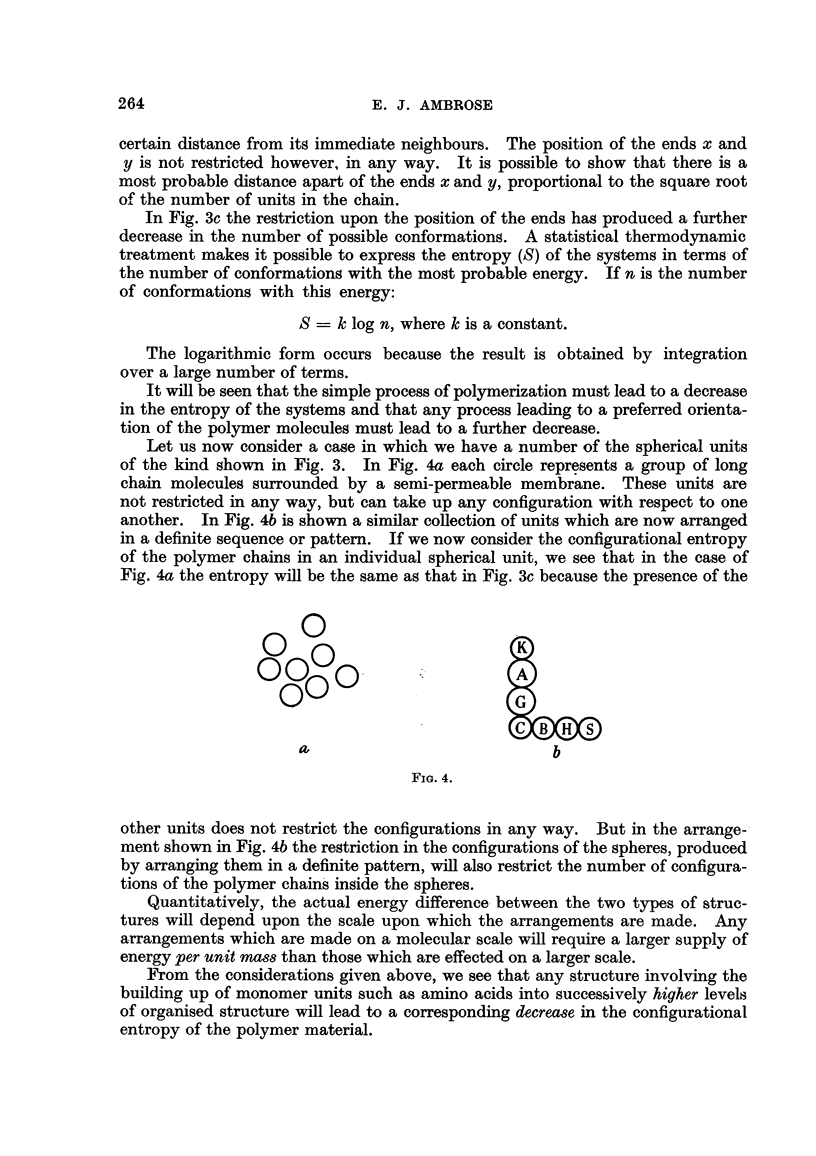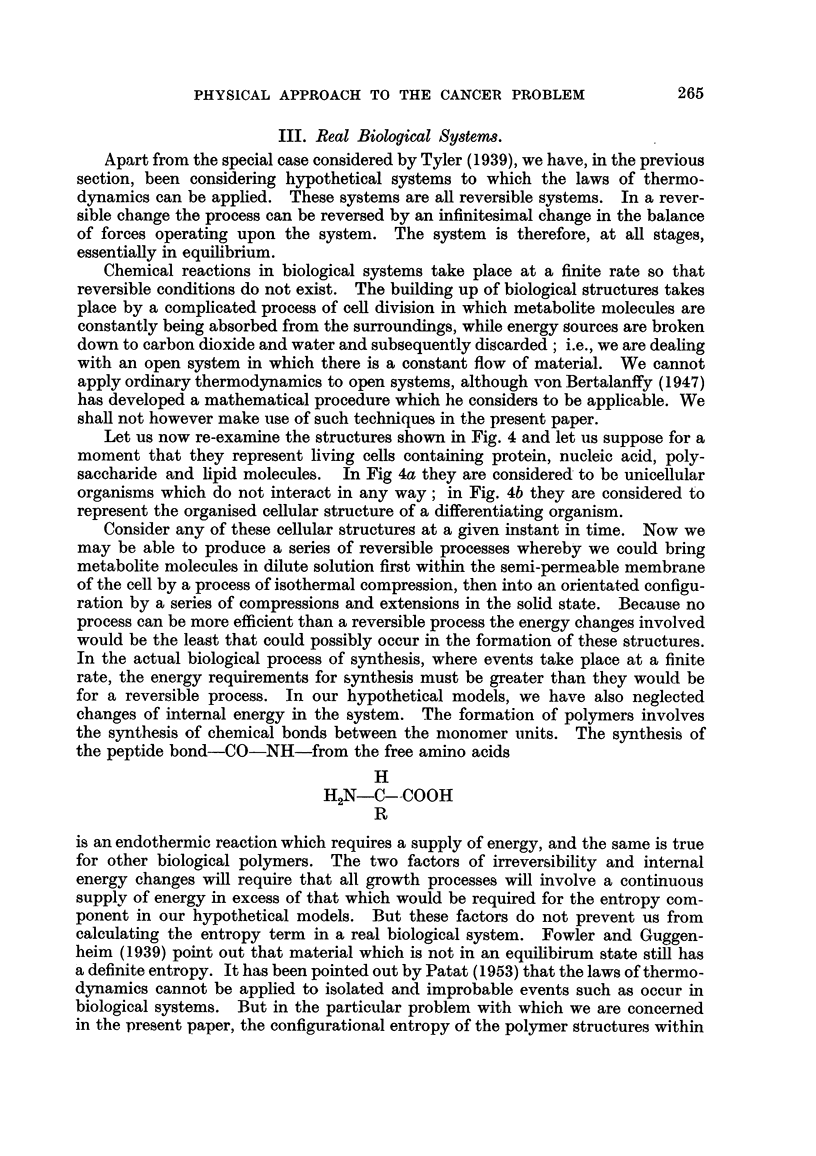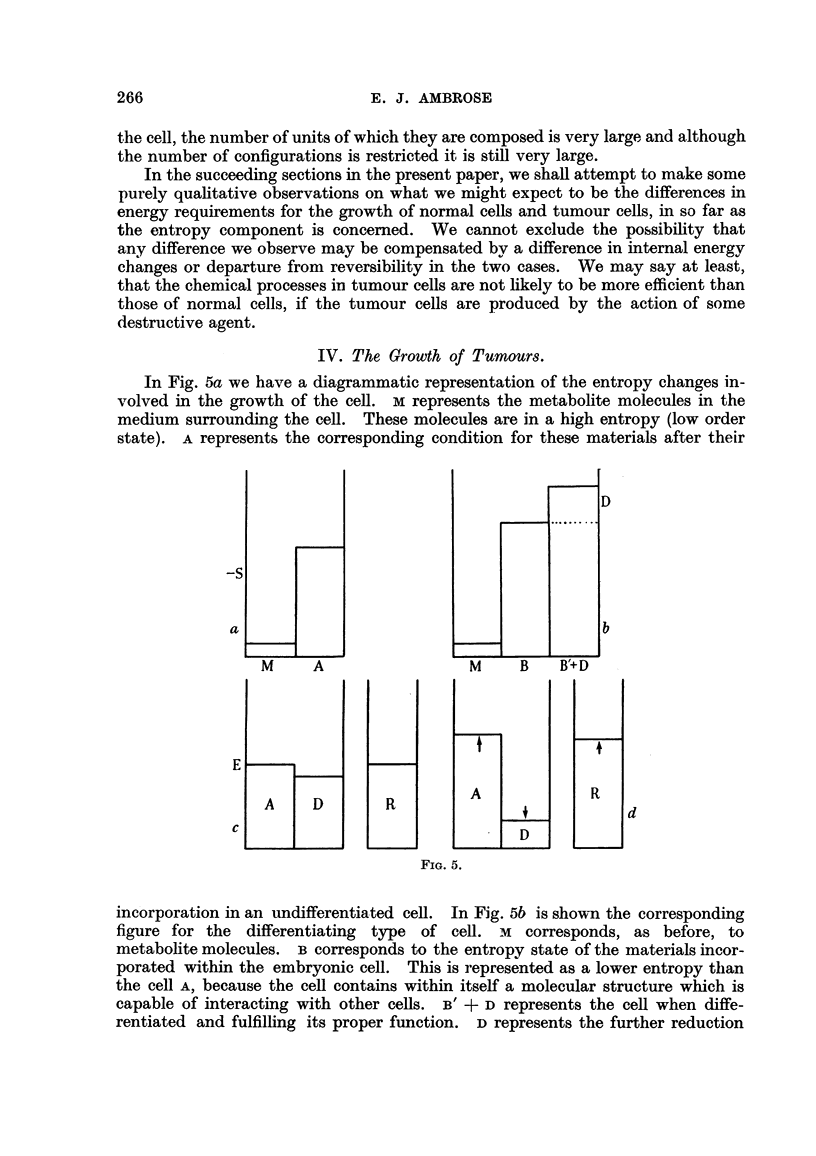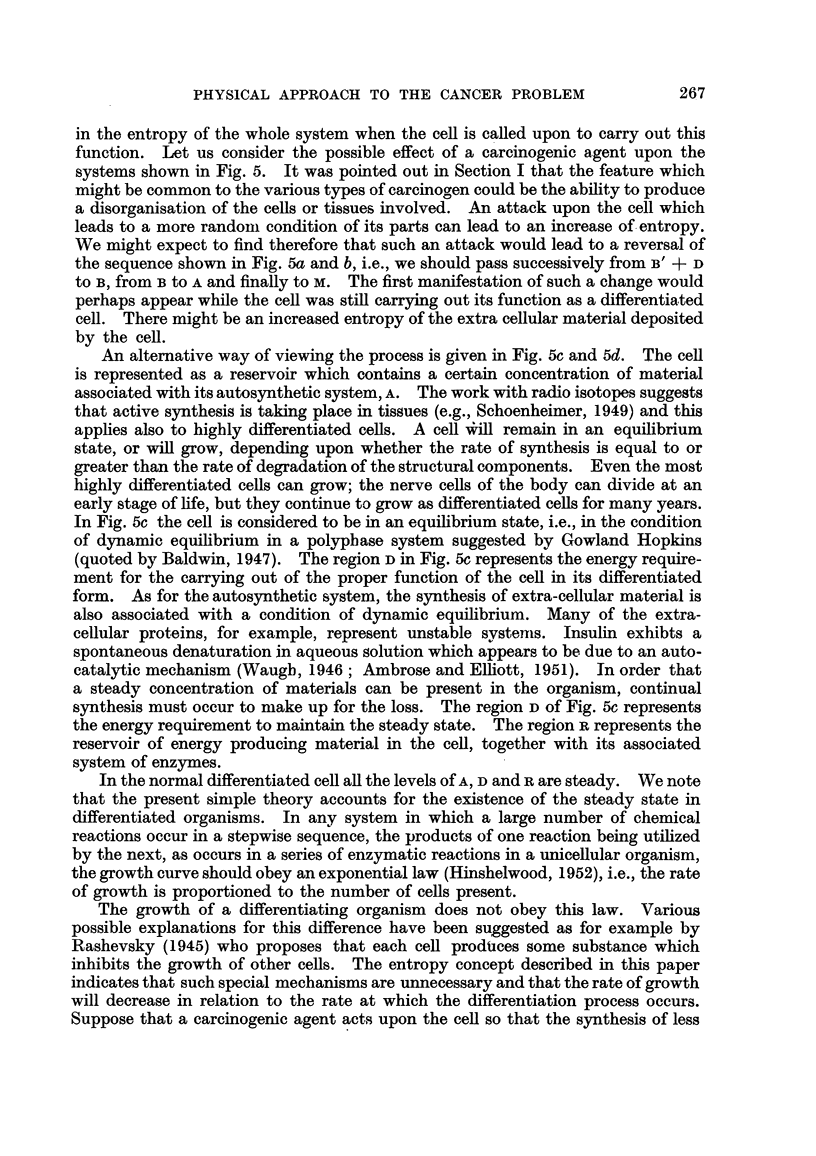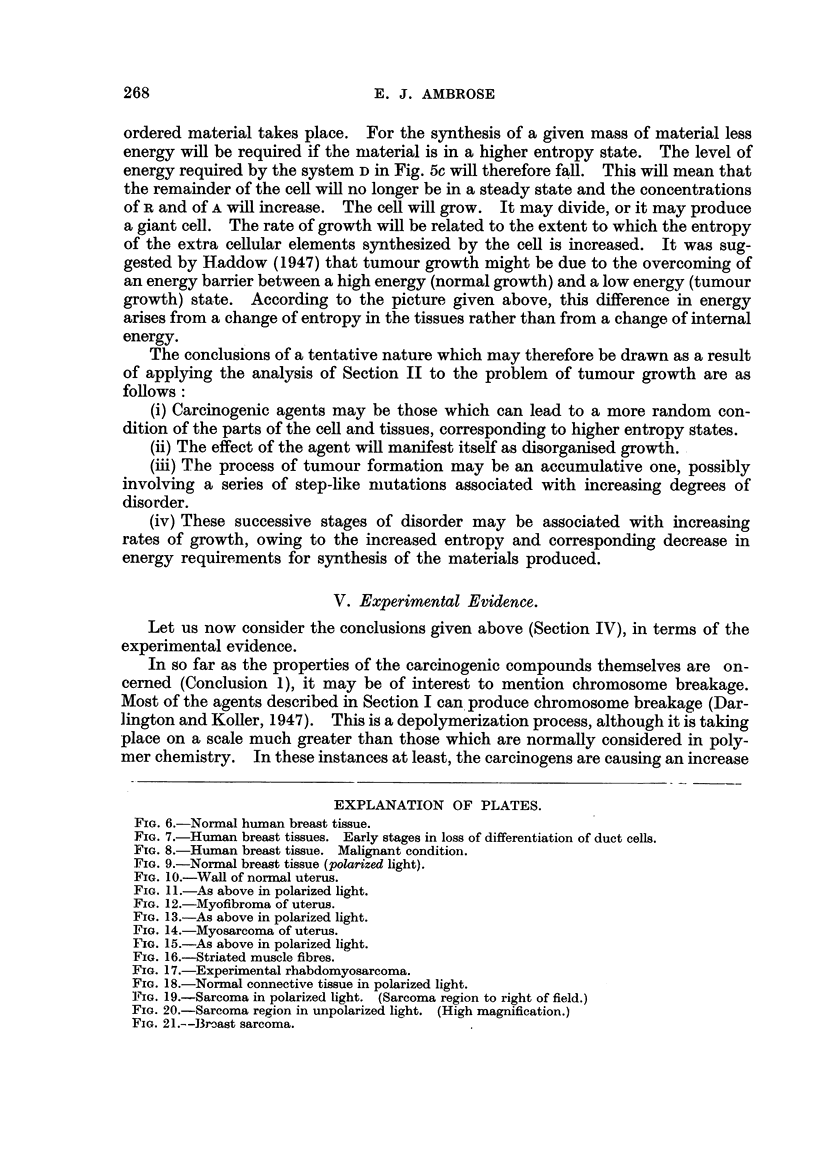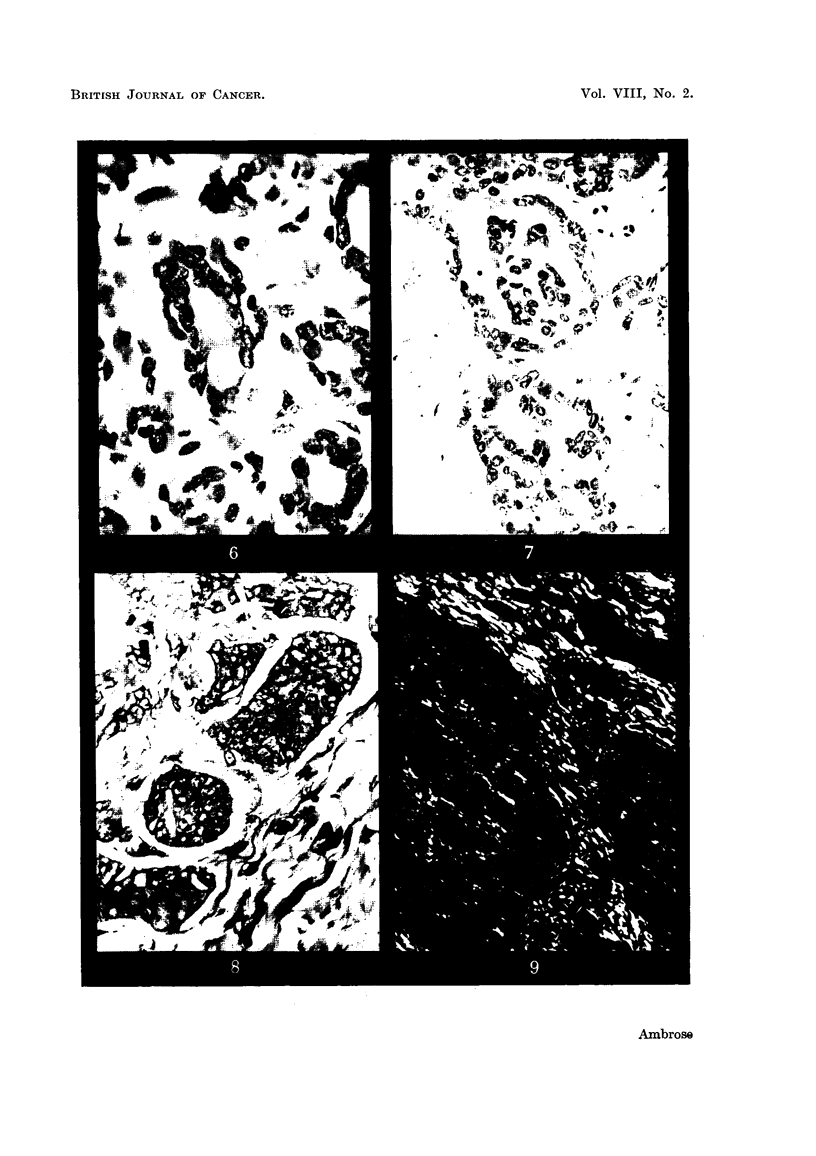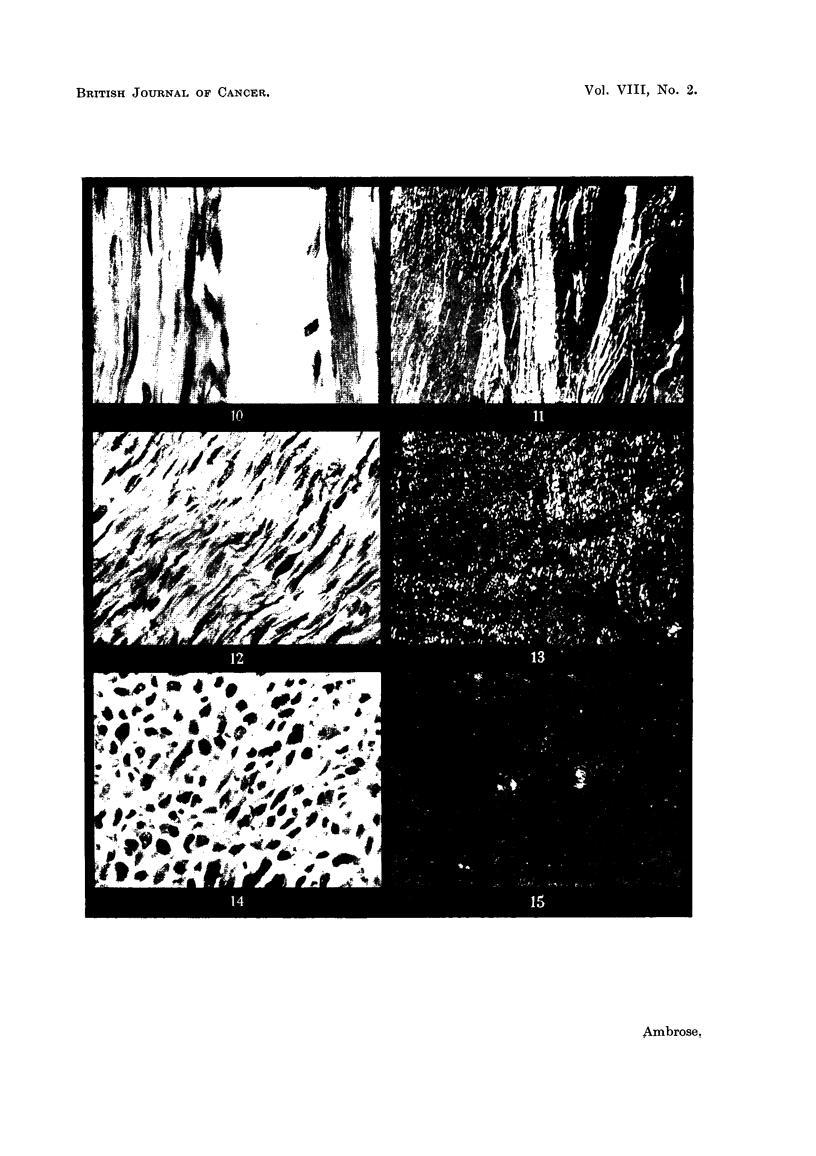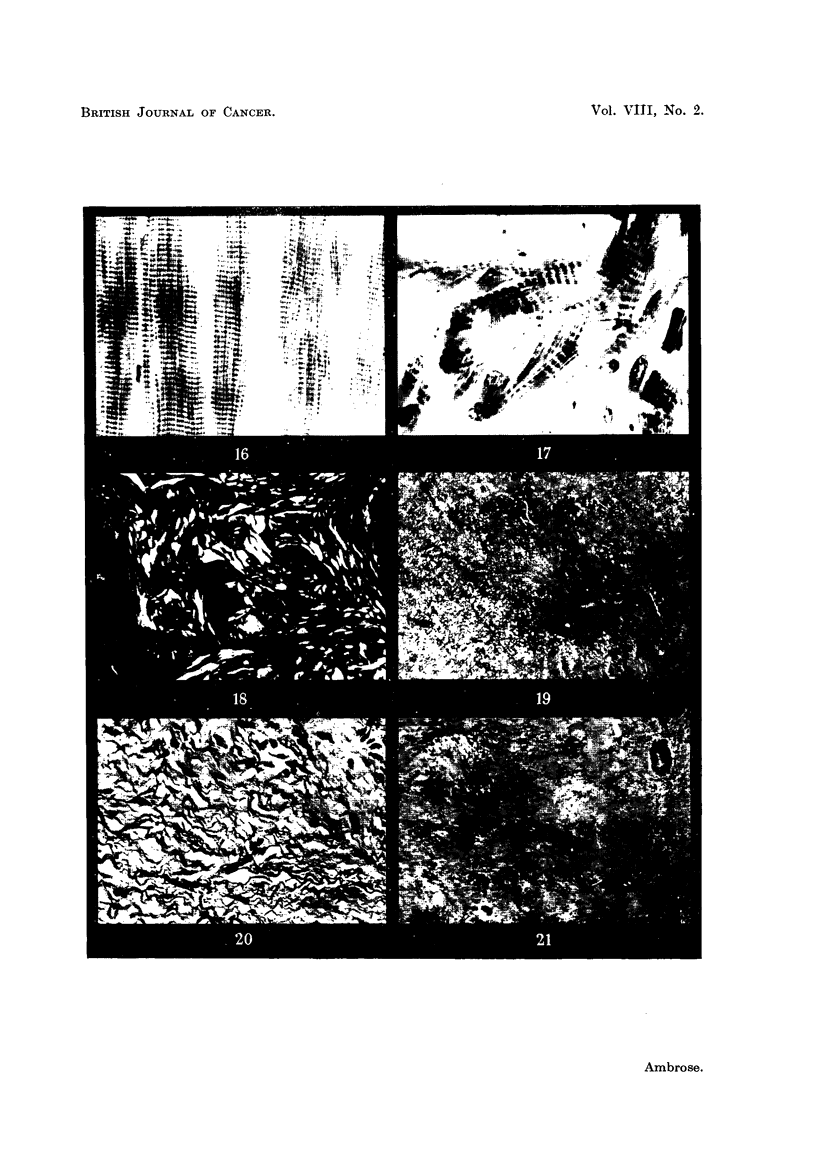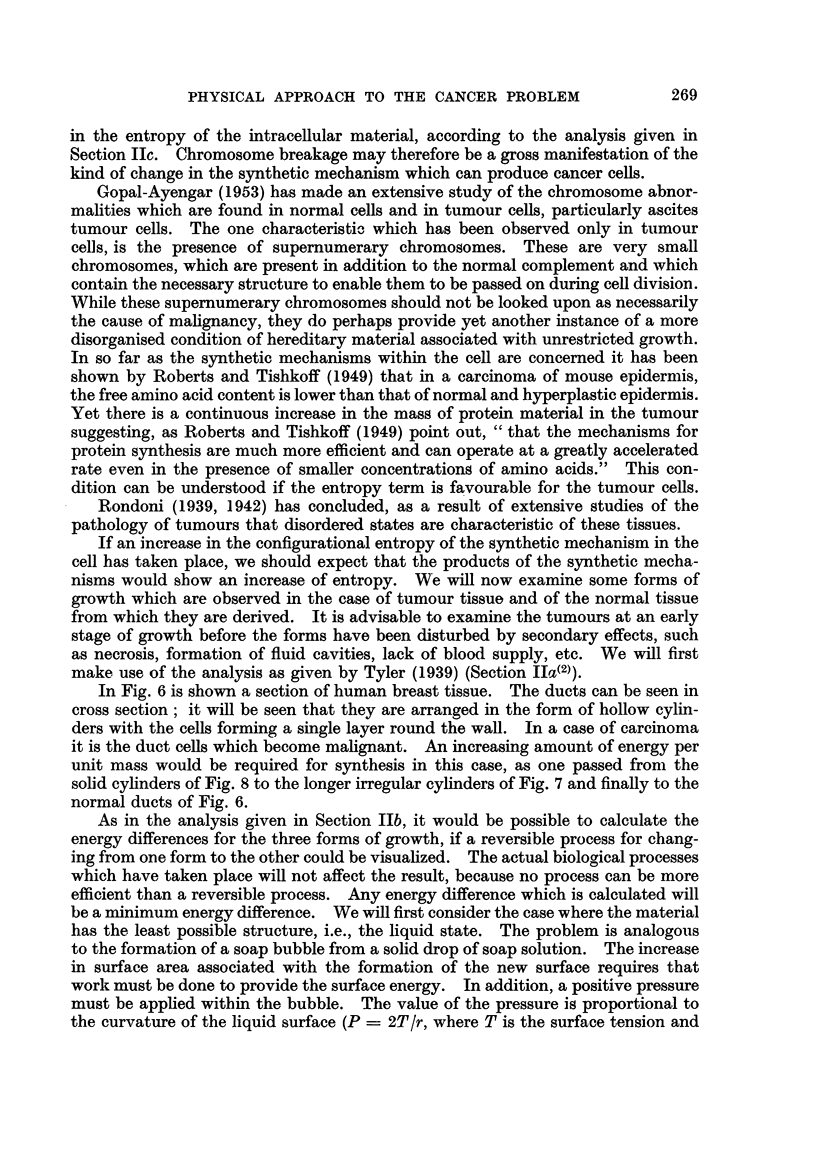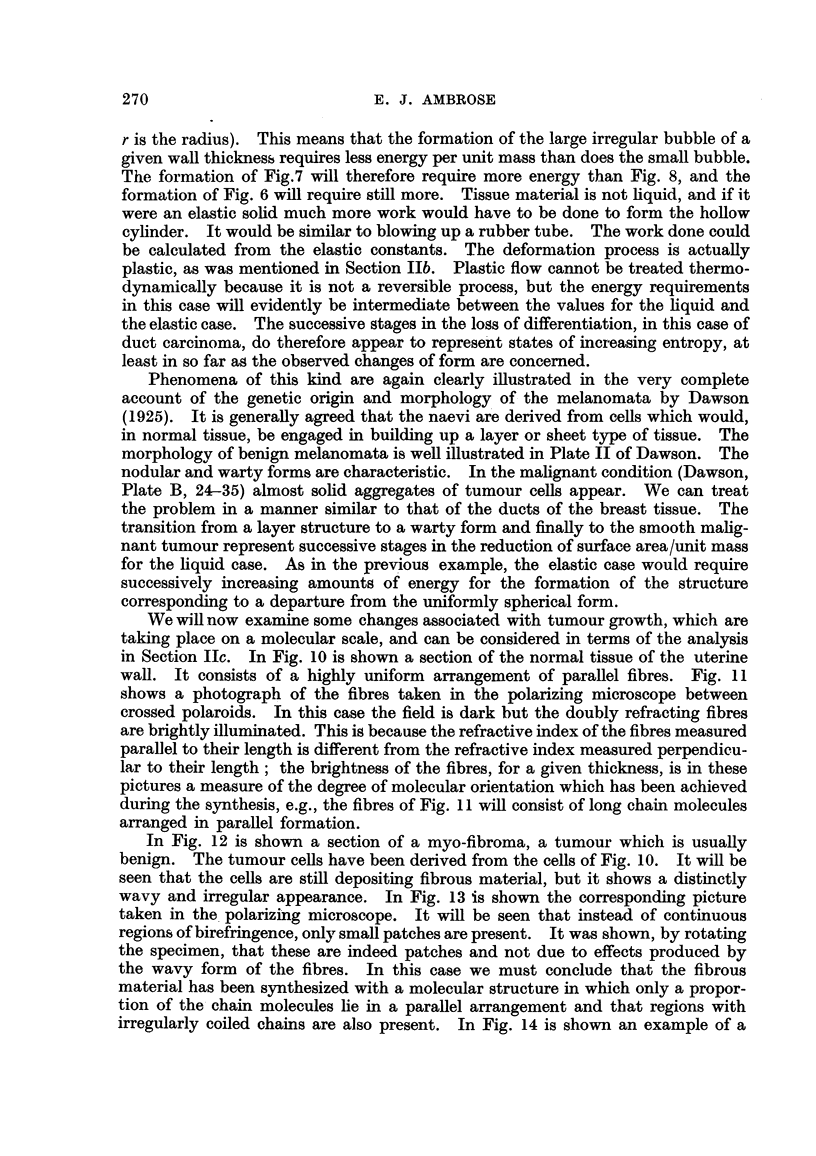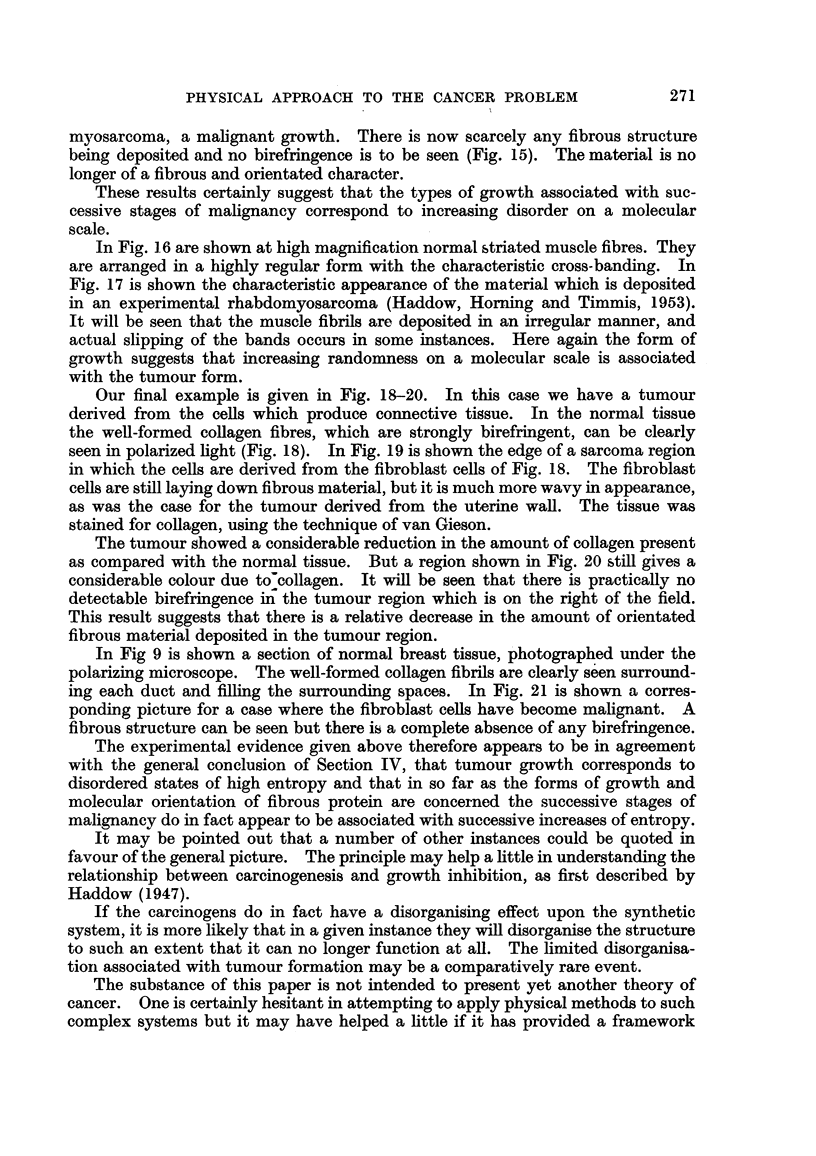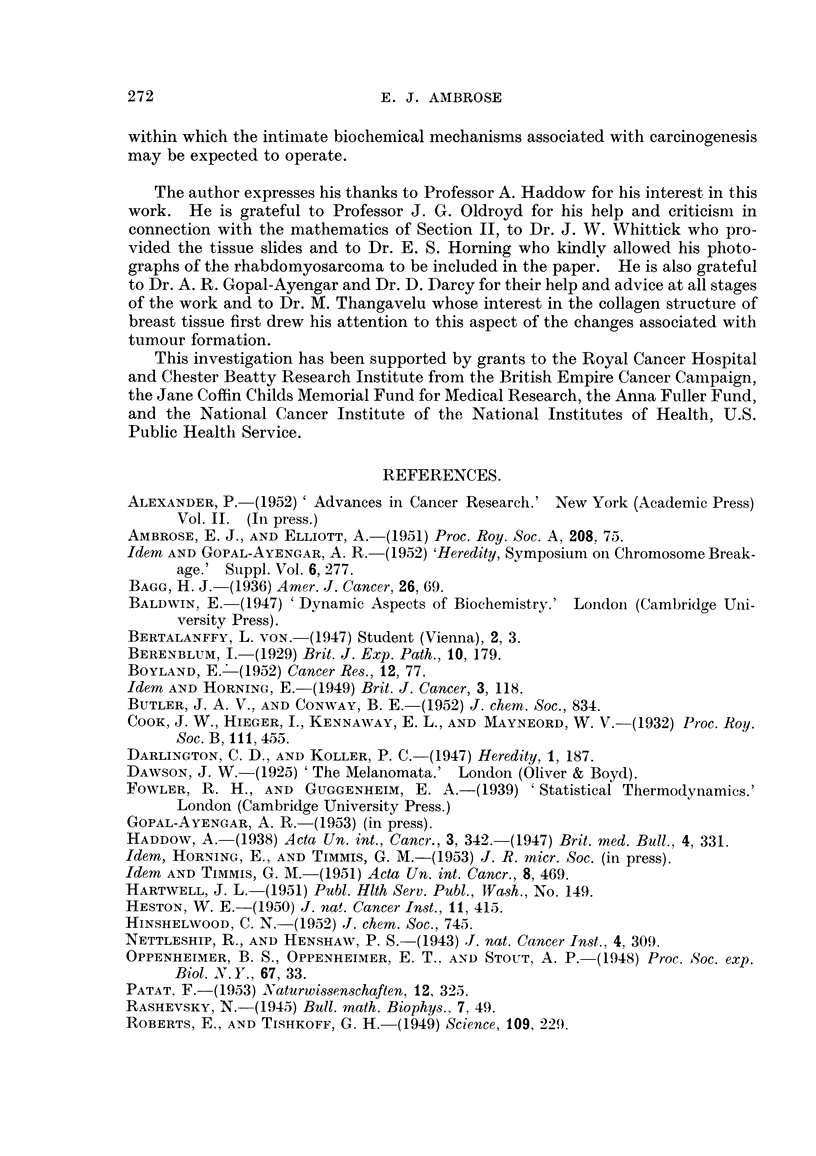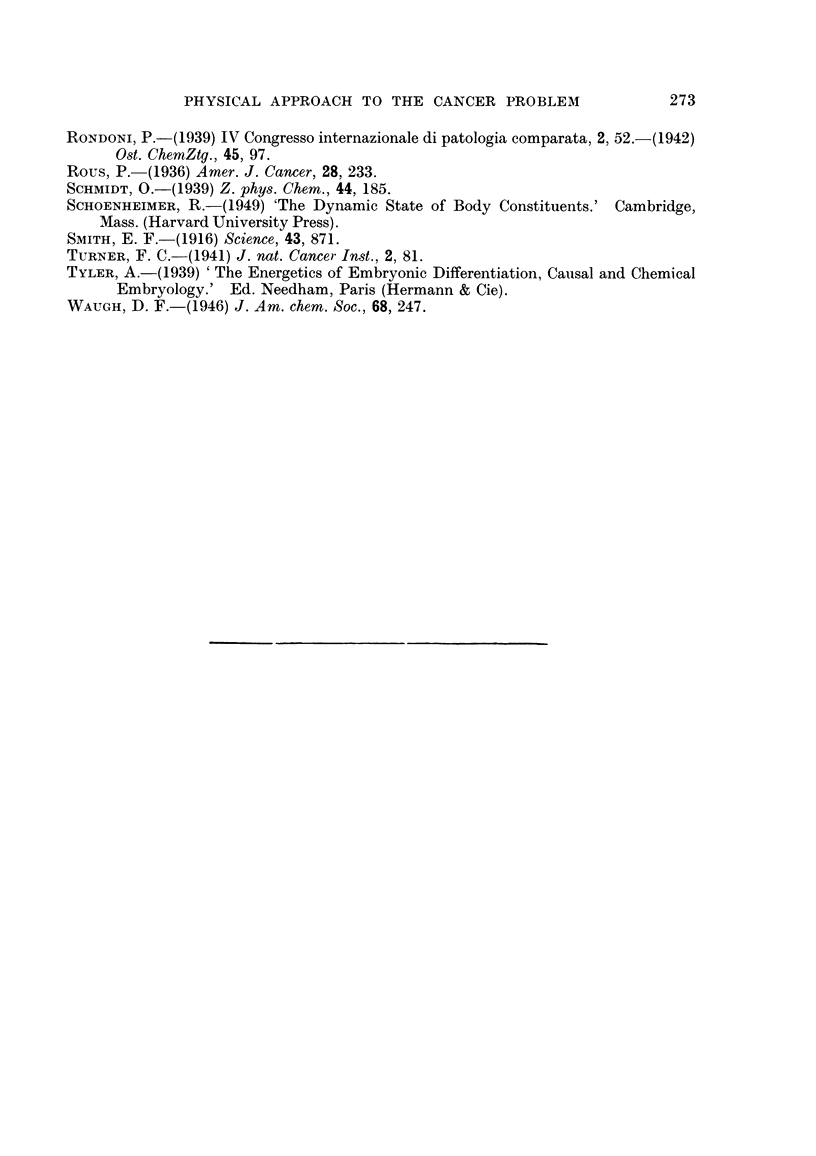# A Physical Approach to the Problem of Cancer

**DOI:** 10.1038/bjc.1954.27

**Published:** 1954-06

**Authors:** E. J. Ambrose

## Abstract

**Images:**


### A PHYSICAL APPROACH TO THE PROBLEM OF CANCER.

E. J. AMBROSE.

*From the Chester Beatty Research Institute, Royal Cancer Hospital, Fulham Road, London, S.W.3.*

Received for publication April 26, 1954.

THE changes which occur in the molecular structure of cells, when a tissue changes from a benign to a malignant form, appear to be of such a kind that they cannot be analysed by present physical or chemical techniques. While this may be true for the problem on a molecular scale, it does not follow, *ipso facto*, that an investigation cannot be made at a higher level of organisation. Hinshelwood (1952) has pointed out, for example, that the three-body problem in mechanics cannot be solved, although the kinetic theory can explain the behaviour of a large number of bodies. He has shown that bacterial growth can be understood in terms of certain simple mathematical relationships, in spite of the fact that the molecular mechanisms involved are extraordinarily complicated.

In this paper an attempt is made to see if such an approach may help to throw a little light on the problem of tumour formation and growth. A brief review is first given of the various physical, chemical and biological methods of producing tumours. This is followed by a mathematical analysis of the problems of growth and differentiation (Sections II and III); an attempt is then made to apply the methods to tumour formation and finally some experimental evidence is given to support the general conclusions.

### I. *A Brief Review of Carcinogenesis.*

The various ways of producing cancer have been reviewed in a recent paper by Boyland (1952). But it will help for the purposes of the present communication to provide a summary of the results.

(a) *Chemical carcinogens.*—The first chemical compound to be identified as a carcinogenic agent was 1:2:5:6-dibenzanthracene (Cook, Hieger, Kennaway and Mayneord, 1932). Since that time, the polycyclic hydrocarbons have been investigated systematically from this point of view. A certain degree of specificity has been observed and it has been correlated to some extent with the presence of a region of high electron density (*K* region, Schmidt, 1939), within the molecule. Some recent work has suggested, however, that the carcinogenic activity of the polycyclic hydrocarbons may be related in part to their ability to undergo auto-oxidation and liberate free radicals (Alexander, 1952).

The second class of carcinogenic compounds includes mustard gas (Heston, 1950), nitrogen mustards (Boyland and Horning, 1949), epoxides, mesyl glycols (Haddow and Timmis, 1951), etc. These compounds are all known to act as alkylating or esterifying agents and probably produce their effects on the body by this type of reaction.

Certain inorganic salts of beryllium and zinc can produce tumours (Bagg, 1936). There are also a number of other chemical compounds, showing little relationship to those mentioned above, which can induce the formation of tumours. Urethane (Nettleship and Henshaw, 1943) is an example of this kind. It might, from the nature of its amide group be expected to attack the secondary forces of cohesion known as hydrogen bonds, which play a most important part in maintaining the structure of biological organelles such as chromosomes (Ambrose and Gopal-Ayengar, 1952).

(b) *Physical methods of producing cancer.*—It appears that the simple process of implanting an inert object such as a disc of bakelite (Turner, 1941) or sheets of cellophane (Oppenheimer, Oppenheimer and Stout, 1948) into a tissue can produce a tumour, while an extreme change of temperature produced by the application of carbon dioxide snow (Berenblum, 1929) can have a similar effect.

Although the ionising radiations can induce cancer, it is probable that their mode of action is indirect and is due to the formation of free radicals (Butler and Conway, 1952).

(c) *Biological carcinogens.*—The well-known Rous sarcoma can be propagated by virus particles (Rous, 1936). Certain parasitic organisms are able to cause a malignant change to occur in the tissue (e.g., Phytomonas tumefaciens in plants, Smith, 1916). The spontaneous tumours, whose origins are unknown should perhaps be considered to be due to the action of a biological carcinogen.

It was pointed out by Haddow in 1938 that there was a striking multiplicity of tumour-producing agents and, as can be seen from the summary given above, the number has greatly increased since that time (Hartwell, 1951). The existence of so many agents suggests, at first sight, that we are dealing with a problem of baffling complexity. But there is just a possibility that the great variety may itself provide a clue as to the mechanisms involved. The agents appear to have one feature in common ; they may be expected to lead to a more random configuration of the molecular structure within the cell, than is present in the normal tissue. We will consider the effects which a change of this kind might be expected to produce upon the economy and energy requirements of the cell.

### II. *A Mathematical Approach to the Problems of Growth and Differentiation.*

We will first attempt to consider problems of growth and differentiation in terms of certain simple models, to which the laws of thermodynamics can be applied. We will try, at a later stage, to see if the models may have any bearing on the behaviour of real biological systems.

### (a) *Classical treatment.*

(1) *Simple growth.*—We will consider the very simple system shown in Fig. 1. The sphere of radius $r_1$ is considered to contain a dilute solution of metabolite molecules ; the molecules could, for example, be molecules of amino acids. It will be assumed that the solution is ideal ; i.e., that the molecules are quite independent of each other and do not interact with the solvent molecules. They behave like the molecules of an inert gas. Suppose that the molecules are surrounded by a hypothetical semi-permeable membrane, which allows free passage for the solvent but not for the metabolite molecules. If the metabolite molecules are compressed reversibly into the volume of the sphere of radius $r_2$ at constant temperature, an amount of work equal to $nRT \log V_1/V_2$ must be done for a fraction $n$ of metabolite molecules, where $n$ is the molar fraction of molecules of this species. If there are other species present, these will require corresponding amounts of energy and the total will be the sum of all components $\Sigma n_r RT \log V_1/V_2$.

Because no process can require less energy than a reversible process it follows that it is necessary to expend energy in this way in all cases of growth. The work which has to be done is generally known as osmotic work. In thermodynamic nomenclature the work which must be done on the system is in this case expressed as a change of entropy. The entropy of any system can be expressed as a measure of the degree of order or disorder which may be present, *a low entropy* corresponding to a *high state of order*. In the case described above, the growth process has involved a decrease of entropy.

Other thermodynamic concepts such as free energy changes are commonly used in studying chemical equilibria, but in this case we shall be concerned with the shape or microscopic morphology of biological material and it will be found that the entropy concept is more useful.

FIG. 1.

In real systems the energy required for the decrease of entropy is in some cases balanced by a change in the potential energy, e.g., the formation of a chemical precipitate is accompanied by a decrease of potential energy, because forces of attraction occur between the molecules forming the precipitate and a closer approach of the molecules leads to a decrease of potential energy.

(2) *Growth with differentiation.*—We will at this stage consider the problem of growth with differentiation since it has been the subject of a beautiful study by Tyler (1939), using sea urchin eggs, although the treatment is not in this case strictly thermodynamic.

The cells at the two cell stage of embryonic development are isolated. They will each continue to develop but they will not develop as the normal embryos. They contain half as many cells as the normal embryo at corresponding stages of development.

In Fig. 2*a* is shown a normal embryo at the blastomere stage of development. The external radius is $R$ units and the wall thickness is $d$ units. There is a single layer of cells forming the wall thickness. In Fig. 2*c* is shown a dwarf embryo, in which it is found experimentally that the surface area is half that of the normal embryo. The radius is therefore $R/\sqrt{2}$ units. The wall thickness, which is the thickness of a single cell, is the same as that of the normal embryo ($d$ units).

At this stage of development invagination begins to take place as shown in Fig. 2*d*.

It is possible to compare the energy requirements for growth in the case of the normal and dwarf embryos by making use of the principle of dimensional analysis (Gallileo, Rayleigh, Tolman, Buckingham). These principles tell us that there is a relationship between all physical quantities and the dimensions in which they are expressed, so that by making use of this principle, it is possible to calculate the magnitude of any quantity, for a case on a different scale of dimensions from those originally chosen.

Alternatively, we may say that in a universe which was constructed on a different scale, e.g., with its linear dimensions half those of the present universe, we should find that this 1:2 relationship would apply also to the other physical quantities, which can be expressed in terms of mass length and time.

In Fig. 2*b* is shown a hypothetical dwarf embryo in which all the dimensions have been reduced in the ratio of $1:1/\sqrt{2}$. The wall thickness is $D/\sqrt{2}$ units and

FIG. 2.
(In *d* the longer line between arrows indicates r.)

not *d* units as in the real dwarf embryo shown in Fig. 2*c*. Suppose that an invagination of the wall of the embryo takes place to the extent of *a* units in the normal embryo at gastrulation. Then an invagination of $a/\sqrt{2}$ units will take place in the hypothetical dwarf.

A process of deformation of the kind shown in Fig. 2*d* will require work, which is measured as a product of the applied force ($F$) and the distance moved ($a$), in the case of the normal embryo. There are two possible ways in which the deformation can take place. It can be either as a plastic or as an elastic deformation. Tyler (1939) considers that the deformation is plastic for a number of reasons, the main one being the fact that organisms do not tend to return to a spherical shape when the cells die.

For the plastic case, in which the work done is proportional to the square of the linear dimensions, the work is equal to $F.a$ units. In the case of the hypothetical dwarf, it will be, according to the methods of dimensional analysis, equal to $F/\sqrt{2}\times a/\sqrt{2} = F.a/2$.

In the case of the real dwarf the thickness of the wall is $d$ and not $d/\sqrt{2}$. If the deformation is plastic the force required will be a linear function of the thickness and will be equal to $F/\sqrt{2}\times\dfrac{d}{d/\sqrt{2}} = F$. The work done will be $F.a/\sqrt{2}$. The two dwarfs together will require an amount of work equal to $2\times F.a/\sqrt{2}$ to be done, i.e., $F.a\sqrt{2}$ or 1·41 times as much as the normal embryo. It can be shown mathematically that if the deformation is elastic the work done will vary as the cube of the linear dimensions and the difference between the energy requirements of the normal and dwarf embryos would be correspondingly increased.

It is found by experiment that the normal and dwarf embryos respire at the same rate but that there is a retardation in the rate of development of the dwarf embryos as compared with the normal of between 30 and 40 per cent. This means that the dwarf embryos require 30–40 per cent more energy for the differentiation process. The agreement with the theoretical prediction is good and signifies that the deformations involved in the growth process are probably largely plastic. If the deformations were elastic a considerably greater difference between the energy requirements would be expected in the two cases.

We see from these results that differentiation is an energy absorbing process.

(b) *Statistical mechanical treatment.*

The treatment given above, which is due mainly to Tyler (1939), represents a study of differentiation in terms of the tissues of the organism involved. We will now attempt an analysis of the problem on a molecular scale with hypothetical models, making use of known data concerning the physics of long chain molecules. High polymers provide the basic structural elements of all living systems, so that growth and differentiation are intimately connected with changes in such systems. As a preliminary treatment, we will neglect all changes of internal energy which may be involved in any process of polymerization or alteration in the configuration of the molecules.

*a* *b* *c*

FIG. 3.

In Fig. 3*a* the metabolite molecules, of the kind which were described in Section II*a*, are represented as rod-like particles. They could for example be simplified molecules of amino acids. They are considered to be confined within the sphere of radius $r_2$ by a semi-permeable membrane, but they are not restrained in any other way. As before, they are considered to behave like the molecules of an inert gas. In Fig. 3*b* the molecules are shown to be arranged in a linear sequence, in the form of a polymer chain. In this case the chain is coiled in an irregular manner. In Fig. 3*c* the chain has been stretched, as it would be in the case of a piece of stretched rubber.

Let us consider the number of possible arrangements or conformations, as they are called, in the three figures. In Fig. 3*a* there are very few restrictions upon the arrangement of the metabolite molecules. It is only necessary that one molecule should not coincide with another and that they should all be confined within the sphere of radius $r$. In Fig. 3*b* we see that the number of possible ways of arranging the units is greatly reduced, because each unit must be maintained at a certain distance from its immediate neighbours. The position of the ends $x$ and $y$ is not restricted however, in any way. It is possible to show that there is a most probable distance apart of the ends $x$ and $y$, proportional to the square root of the number of units in the chain.

In Fig. 3*c* the restriction upon the position of the ends has produced a further decrease in the number of possible conformations. A statistical thermodynamic treatment makes it possible to express the entropy ($S$) of the systems in terms of the number of conformations with the most probable energy. If $n$ is the number of conformations with this energy:

$$S = k \log n, \text{ where } k \text{ is a constant.}$$

The logarithmic form occurs because the result is obtained by integration over a large number of terms.

It will be seen that the simple process of polymerization must lead to a decrease in the entropy of the systems and that any process leading to a preferred orientation of the polymer molecules must lead to a further decrease.

Let us now consider a case in which we have a number of the spherical units of the kind shown in Fig. 3. In Fig. 4*a* each circle represents a group of long chain molecules surrounded by a semi-permeable membrane. These units are not restricted in any way, but can take up any configuration with respect to one another. In Fig. 4*b* is shown a similar collection of units which are now arranged in a definite sequence or pattern. If we now consider the configurational entropy of the polymer chains in an individual spherical unit, we see that in the case of Fig. 4*a* the entropy will be the same as that in Fig. 3*c* because the presence of the

*a* *b*

FIG. 4.

other units does not restrict the configurations in any way. But in the arrangement shown in Fig. 4*b* the restriction in the configurations of the spheres, produced by arranging them in a definite pattern, will also restrict the number of configurations of the polymer chains inside the spheres.

Quantitatively, the actual energy difference between the two types of structures will depend upon the scale upon which the arrangements are made. Any arrangements which are made on a molecular scale will require a larger supply of energy *per unit mass* than those which are effected on a larger scale.

From the considerations given above, we see that any structure involving the building up of monomer units such as amino acids into successively *higher* levels of organised structure will lead to a corresponding *decrease* in the configurational entropy of the polymer material.

### III. *Real Biological Systems.*

Apart from the special case considered by Tyler (1939), we have, in the previous section, been considering hypothetical systems to which the laws of thermodynamics can be applied. These systems are all reversible systems. In a reversible change the process can be reversed by an infinitesimal change in the balance of forces operating upon the system. The system is therefore, at all stages, essentially in equilibrium.

Chemical reactions in biological systems take place at a finite rate so that reversible conditions do not exist. The building up of biological structures takes place by a complicated process of cell division in which metabolite molecules are constantly being absorbed from the surroundings, while energy sources are broken down to carbon dioxide and water and subsequently discarded ; i.e., we are dealing with an open system in which there is a constant flow of material. We cannot apply ordinary thermodynamics to open systems, although von Bertalanffy (1947) has developed a mathematical procedure which he considers to be applicable. We shall not however make use of such techniques in the present paper.

Let us now re-examine the structures shown in Fig. 4 and let us suppose for a moment that they represent living cells containing protein, nucleic acid, polysaccharide and lipid molecules. In Fig 4*a* they are considered to be unicellular organisms which do not interact in any way ; in Fig. 4*b* they are considered to represent the organised cellular structure of a differentiating organism.

Consider any of these cellular structures at a given instant in time. Now we may be able to produce a series of reversible processes whereby we could bring metabolite molecules in dilute solution first within the semi-permeable membrane of the cell by a process of isothermal compression, then into an orientated configuration by a series of compressions and extensions in the solid state. Because no process can be more efficient than a reversible process the energy changes involved would be the least that could possibly occur in the formation of these structures. In the actual biological process of synthesis, where events take place at a finite rate, the energy requirements for synthesis must be greater than they would be for a reversible process. In our hypothetical models, we have also neglected changes of internal energy in the system. The formation of polymers involves the synthesis of chemical bonds between the monomer units. The synthesis of the peptide bond—CO—NH—from the free amino acids

$$\begin{array}{c} \mathrm{H} \\ \mathrm{H_2N{-}C{-}COOH} \\ \mathrm{R} \end{array}$$

is an endothermic reaction which requires a supply of energy, and the same is true for other biological polymers. The two factors of irreversibility and internal energy changes will require that all growth processes will involve a continuous supply of energy in excess of that which would be required for the entropy component in our hypothetical models. But these factors do not prevent us from calculating the entropy term in a real biological system. Fowler and Guggenheim (1939) point out that material which is not in an equilibirum state still has a definite entropy. It has been pointed out by Patat (1953) that the laws of thermodynamics cannot be applied to isolated and improbable events such as occur in biological systems. But in the particular problem with which we are concerned in the present paper, the configurational entropy of the polymer structures within the cell, the number of units of which they are composed is very large and although the number of configurations is restricted it is still very large.

In the succeeding sections in the present paper, we shall attempt to make some purely qualitative observations on what we might expect to be the differences in energy requirements for the growth of normal cells and tumour cells, in so far as the entropy component is concerned. We cannot exclude the possibility that any difference we observe may be compensated by a difference in internal energy changes or departure from reversibility in the two cases. We may say at least, that the chemical processes in tumour cells are not likely to be more efficient than those of normal cells, if the tumour cells are produced by the action of some destructive agent.

### IV. *The Growth of Tumours.*

In Fig. 5*a* we have a diagrammatic representation of the entropy changes involved in the growth of the cell. M represents the metabolite molecules in the medium surrounding the cell. These molecules are in a high entropy (low order state). A represents the corresponding condition for these materials after their

FIG. 5.

incorporation in an undifferentiated cell. In Fig. 5*b* is shown the corresponding figure for the differentiating type of cell. M corresponds, as before, to metabolite molecules. B corresponds to the entropy state of the materials incorporated within the embryonic cell. This is represented as a lower entropy than the cell A, because the cell contains within itself a molecular structure which is capable of interacting with other cells. B′ + D represents the cell when differentiated and fulfilling its proper function. D represents the further reduction in the entropy of the whole system when the cell is called upon to carry out this function. Let us consider the possible effect of a carcinogenic agent upon the systems shown in Fig. 5. It was pointed out in Section I that the feature which might be common to the various types of carcinogen could be the ability to produce a disorganisation of the cells or tissues involved. An attack upon the cell which leads to a more random condition of its parts can lead to an increase of entropy. We might expect to find therefore that such an attack would lead to a reversal of the sequence shown in Fig. 5*a* and *b*, i.e., we should pass successively from B′ + D to B, from B to A and finally to M. The first manifestation of such a change would perhaps appear while the cell was still carrying out its function as a differentiated cell. There might be an increased entropy of the extra cellular material deposited by the cell.

An alternative way of viewing the process is given in Fig. 5*c* and 5*d*. The cell is represented as a reservoir which contains a certain concentration of material associated with its autosynthetic system, A. The work with radio isotopes suggests that active synthesis is taking place in tissues (e.g., Schoenheimer, 1949) and this applies also to highly differentiated cells. A cell will remain in an equilibrium state, or will grow, depending upon whether the rate of synthesis is equal to or greater than the rate of degradation of the structural components. Even the most highly differentiated cells can grow; the nerve cells of the body can divide at an early stage of life, but they continue to grow as differentiated cells for many years. In Fig. 5*c* the cell is considered to be in an equilibrium state, i.e., in the condition of dynamic equilibrium in a polyphase system suggested by Gowland Hopkins (quoted by Baldwin, 1947). The region D in Fig. 5*c* represents the energy requirement for the carrying out of the proper function of the cell in its differentiated form. As for the autosynthetic system, the synthesis of extra-cellular material is also associated with a condition of dynamic equilibrium. Many of the extra-cellular proteins, for example, represent unstable systems. Insulin exhibts a spontaneous denaturation in aqueous solution which appears to be due to an auto-catalytic mechanism (Waugh, 1946 ; Ambrose and Elliott, 1951). In order that a steady concentration of materials can be present in the organism, continual synthesis must occur to make up for the loss. The region D of Fig. 5*c* represents the energy requirement to maintain the steady state. The region R represents the reservoir of energy producing material in the cell, together with its associated system of enzymes.

In the normal differentiated cell all the levels of A, D and R are steady. We note that the present simple theory accounts for the existence of the steady state in differentiated organisms. In any system in which a large number of chemical reactions occur in a stepwise sequence, the products of one reaction being utilized by the next, as occurs in a series of enzymatic reactions in a unicellular organism, the growth curve should obey an exponential law (Hinshelwood, 1952), i.e., the rate of growth is proportioned to the number of cells present.

The growth of a differentiating organism does not obey this law. Various possible explanations for this difference have been suggested as for example by Rashevsky (1945) who proposes that each cell produces some substance which inhibits the growth of other cells. The entropy concept described in this paper indicates that such special mechanisms are unnecessary and that the rate of growth will decrease in relation to the rate at which the differentiation process occurs. Suppose that a carcinogenic agent acts upon the cell so that the synthesis of less ordered material takes place. For the synthesis of a given mass of material less energy will be required if the material is in a higher entropy state. The level of energy required by the system D in Fig. 5c will therefore fall. This will mean that the remainder of the cell will no longer be in a steady state and the concentrations of R and of A will increase. The cell will grow. It may divide, or it may produce a giant cell. The rate of growth will be related to the extent to which the entropy of the extra cellular elements synthesized by the cell is increased. It was suggested by Haddow (1947) that tumour growth might be due to the overcoming of an energy barrier between a high energy (normal growth) and a low energy (tumour growth) state. According to the picture given above, this difference in energy arises from a change of entropy in the tissues rather than from a change of internal energy.

The conclusions of a tentative nature which may therefore be drawn as a result of applying the analysis of Section II to the problem of tumour growth are as follows :

(i) Carcinogenic agents may be those which can lead to a more random condition of the parts of the cell and tissues, corresponding to higher entropy states.

(ii) The effect of the agent will manifest itself as disorganised growth.

(iii) The process of tumour formation may be an accumulative one, possibly involving a series of step-like mutations associated with increasing degrees of disorder.

(iv) These successive stages of disorder may be associated with increasing rates of growth, owing to the increased entropy and corresponding decrease in energy requirements for synthesis of the materials produced.

### V. *Experimental Evidence.*

Let us now consider the conclusions given above (Section IV), in terms of the experimental evidence.

In so far as the properties of the carcinogenic compounds themselves are oncerned (Conclusion 1), it may be of interest to mention chromosome breakage. Most of the agents described in Section I can produce chromosome breakage (Darlington and Koller, 1947). This is a depolymerization process, although it is taking place on a scale much greater than those which are normally considered in polymer chemistry. In these instances at least, the carcinogens are causing an increase

---

EXPLANATION OF PLATES.

FIG. 6.—Normal human breast tissue.
FIG. 7.—Human breast tissues. Early stages in loss of differentiation of duct cells.
FIG. 8.—Human breast tissue. Malignant condition.
FIG. 9.—Normal breast tissue (*polarized* light).
FIG. 10.—Wall of normal uterus.
FIG. 11.—As above in polarized light.
FIG. 12.—Myofibroma of uterus.
FIG. 13.—As above in polarized light.
FIG. 14.—Myosarcoma of uterus.
FIG. 15.—As above in polarized light.
FIG. 16.—Striated muscle fibres.
FIG. 17.—Experimental rhabdomyosarcoma.
FIG. 18.—Normal connective tissue in polarized light.
FIG. 19.—Sarcoma in polarized light. (Sarcoma region to right of field.)
FIG. 20.—Sarcoma region in unpolarized light. (High magnification.)
FIG. 21.—Breast sarcoma.

Ambrose

Ambrose.

16 17 18 19 20 21

Ambrose.

in the entropy of the intracellular material, according to the analysis given in Section II*c*. Chromosome breakage may therefore be a gross manifestation of the kind of change in the synthetic mechanism which can produce cancer cells.

Gopal-Ayengar (1953) has made an extensive study of the chromosome abnormalities which are found in normal cells and in tumour cells, particularly ascites tumour cells. The one characteristic which has been observed only in tumour cells, is the presence of supernumerary chromosomes. These are very small chromosomes, which are present in addition to the normal complement and which contain the necessary structure to enable them to be passed on during cell division. While these supernumerary chromosomes should not be looked upon as necessarily the cause of malignancy, they do perhaps provide yet another instance of a more disorganised condition of hereditary material associated with unrestricted growth. In so far as the synthetic mechanisms within the cell are concerned it has been shown by Roberts and Tishkoff (1949) that in a carcinoma of mouse epidermis, the free amino acid content is lower than that of normal and hyperplastic epidermis. Yet there is a continuous increase in the mass of protein material in the tumour suggesting, as Roberts and Tishkoff (1949) point out, " that the mechanisms for protein synthesis are much more efficient and can operate at a greatly accelerated rate even in the presence of smaller concentrations of amino acids." This condition can be understood if the entropy term is favourable for the tumour cells.

Rondoni (1939, 1942) has concluded, as a result of extensive studies of the pathology of tumours that disordered states are characteristic of these tissues.

If an increase in the configurational entropy of the synthetic mechanism in the cell has taken place, we should expect that the products of the synthetic mechanisms would show an increase of entropy. We will now examine some forms of growth which are observed in the case of tumour tissue and of the normal tissue from which they are derived. It is advisable to examine the tumours at an early stage of growth before the forms have been disturbed by secondary effects, such as necrosis, formation of fluid cavities, lack of blood supply, etc. We will first make use of the analysis as given by Tyler (1939) (Section II$a^{(2)}$).

In Fig. 6 is shown a section of human breast tissue. The ducts can be seen in cross section ; it will be seen that they are arranged in the form of hollow cylinders with the cells forming a single layer round the wall. In a case of carcinoma it is the duct cells which become malignant. An increasing amount of energy per unit mass would be required for synthesis in this case, as one passed from the solid cylinders of Fig. 8 to the longer irregular cylinders of Fig. 7 and finally to the normal ducts of Fig. 6.

As in the analysis given in Section II*b*, it would be possible to calculate the energy differences for the three forms of growth, if a reversible process for changing from one form to the other could be visualized. The actual biological processes which have taken place will not affect the result, because no process can be more efficient than a reversible process. Any energy difference which is calculated will be a minimum energy difference. We will first consider the case where the material has the least possible structure, i.e., the liquid state. The problem is analogous to the formation of a soap bubble from a solid drop of soap solution. The increase in surface area associated with the formation of the new surface requires that work must be done to provide the surface energy. In addition, a positive pressure must be applied within the bubble. The value of the pressure is proportional to the curvature of the liquid surface ($P = 2T/r$, where $T$ is the surface tension and

$r$ is the radius). This means that the formation of the large irregular bubble of a given wall thickness requires less energy per unit mass than does the small bubble. The formation of Fig.7 will therefore require more energy than Fig. 8, and the formation of Fig. 6 will require still more. Tissue material is not liquid, and if it were an elastic solid much more work would have to be done to form the hollow cylinder. It would be similar to blowing up a rubber tube. The work done could be calculated from the elastic constants. The deformation process is actually plastic, as was mentioned in Section II*b*. Plastic flow cannot be treated thermodynamically because it is not a reversible process, but the energy requirements in this case will evidently be intermediate between the values for the liquid and the elastic case. The successive stages in the loss of differentiation, in this case of duct carcinoma, do therefore appear to represent states of increasing entropy, at least in so far as the observed changes of form are concerned.

Phenomena of this kind are again clearly illustrated in the very complete account of the genetic origin and morphology of the melanomata by Dawson (1925). It is generally agreed that the naevi are derived from cells which would, in normal tissue, be engaged in building up a layer or sheet type of tissue. The morphology of benign melanomata is well illustrated in Plate II of Dawson. The nodular and warty forms are characteristic. In the malignant condition (Dawson, Plate B, 24–35) almost solid aggregates of tumour cells appear. We can treat the problem in a manner similar to that of the ducts of the breast tissue. The transition from a layer structure to a warty form and finally to the smooth malignant tumour represent successive stages in the reduction of surface area/unit mass for the liquid case. As in the previous example, the elastic case would require successively increasing amounts of energy for the formation of the structure corresponding to a departure from the uniformly spherical form.

We will now examine some changes associated with tumour growth, which are taking place on a molecular scale, and can be considered in terms of the analysis in Section II*c*. In Fig. 10 is shown a section of the normal tissue of the uterine wall. It consists of a highly uniform arrangement of parallel fibres. Fig. 11 shows a photograph of the fibres taken in the polarizing microscope between crossed polaroids. In this case the field is dark but the doubly refracting fibres are brightly illuminated. This is because the refractive index of the fibres measured parallel to their length is different from the refractive index measured perpendicular to their length ; the brightness of the fibres, for a given thickness, is in these pictures a measure of the degree of molecular orientation which has been achieved during the synthesis, e.g., the fibres of Fig. 11 will consist of long chain molecules arranged in parallel formation.

In Fig. 12 is shown a section of a myo-fibroma, a tumour which is usually benign. The tumour cells have been derived from the cells of Fig. 10. It will be seen that the cells are still depositing fibrous material, but it shows a distinctly wavy and irregular appearance. In Fig. 13 is shown the corresponding picture taken in the polarizing microscope. It will be seen that instead of continuous regions of birefringence, only small patches are present. It was shown, by rotating the specimen, that these are indeed patches and not due to effects produced by the wavy form of the fibres. In this case we must conclude that the fibrous material has been synthesized with a molecular structure in which only a proportion of the chain molecules lie in a parallel arrangement and that regions with irregularly coiled chains are also present. In Fig. 14 is shown an example of a myosarcoma, a malignant growth. There is now scarcely any fibrous structure being deposited and no birefringence is to be seen (Fig. 15). The material is no longer of a fibrous and orientated character.

These results certainly suggest that the types of growth associated with successive stages of malignancy correspond to increasing disorder on a molecular scale.

In Fig. 16 are shown at high magnification normal striated muscle fibres. They are arranged in a highly regular form with the characteristic cross-banding. In Fig. 17 is shown the characteristic appearance of the material which is deposited in an experimental rhabdomyosarcoma (Haddow, Horning and Timmis, 1953). It will be seen that the muscle fibrils are deposited in an irregular manner, and actual slipping of the bands occurs in some instances. Here again the form of growth suggests that increasing randomness on a molecular scale is associated with the tumour form.

Our final example is given in Fig. 18–20. In this case we have a tumour derived from the cells which produce connective tissue. In the normal tissue the well-formed collagen fibres, which are strongly birefringent, can be clearly seen in polarized light (Fig. 18). In Fig. 19 is shown the edge of a sarcoma region in which the cells are derived from the fibroblast cells of Fig. 18. The fibroblast cells are still laying down fibrous material, but it is much more wavy in appearance, as was the case for the tumour derived from the uterine wall. The tissue was stained for collagen, using the technique of van Gieson.

The tumour showed a considerable reduction in the amount of collagen present as compared with the normal tissue. But a region shown in Fig. 20 still gives a considerable colour due to collagen. It will be seen that there is practically no detectable birefringence in the tumour region which is on the right of the field. This result suggests that there is a relative decrease in the amount of orientated fibrous material deposited in the tumour region.

In Fig 9 is shown a section of normal breast tissue, photographed under the polarizing microscope. The well-formed collagen fibrils are clearly seen surrounding each duct and filling the surrounding spaces. In Fig. 21 is shown a corresponding picture for a case where the fibroblast cells have become malignant. A fibrous structure can be seen but there is a complete absence of any birefringence.

The experimental evidence given above therefore appears to be in agreement with the general conclusion of Section IV, that tumour growth corresponds to disordered states of high entropy and that in so far as the forms of growth and molecular orientation of fibrous protein are concerned the successive stages of malignancy do in fact appear to be associated with successive increases of entropy.

It may be pointed out that a number of other instances could be quoted in favour of the general picture. The principle may help a little in understanding the relationship between carcinogenesis and growth inhibition, as first described by Haddow (1947).

If the carcinogens do in fact have a disorganising effect upon the synthetic system, it is more likely that in a given instance they will disorganise the structure to such an extent that it can no longer function at all. The limited disorganisation associated with tumour formation may be a comparatively rare event.

The substance of this paper is not intended to present yet another theory of cancer. One is certainly hesitant in attempting to apply physical methods to such complex systems but it may have helped a little if it has provided a framework within which the intimate biochemical mechanisms associated with carcinogenesis may be expected to operate.

The author expresses his thanks to Professor A. Haddow for his interest in this work. He is grateful to Professor J. G. Oldroyd for his help and criticism in connection with the mathematics of Section II, to Dr. J. W. Whittick who provided the tissue slides and to Dr. E. S. Horning who kindly allowed his photographs of the rhabdomyosarcoma to be included in the paper. He is also grateful to Dr. A. R. Gopal-Ayengar and Dr. D. Darcy for their help and advice at all stages of the work and to Dr. M. Thangavelu whose interest in the collagen structure of breast tissue first drew his attention to this aspect of the changes associated with tumour formation.

This investigation has been supported by grants to the Royal Cancer Hospital and Chester Beatty Research Institute from the British Empire Cancer Campaign, the Jane Coffin Childs Memorial Fund for Medical Research, the Anna Fuller Fund, and the National Cancer Institute of the National Institutes of Health, U.S. Public Health Service.

### REFERENCES.

ALEXANDER, P.—(1952) ' Advances in Cancer Research.' New York (Academic Press) Vol. II. (In press.)

AMBROSE, E. J., AND ELLIOTT, A.—(1951) *Proc. Roy. Soc.* A, **208**, 75.

*Idem* AND GOPAL-AYENGAR, A. R.—(1952) '*Heredity*, Symposium on Chromosome Breakage.' Suppl. Vol. **6**, 277.

BAGG, H. J.—(1936) *Amer. J. Cancer*, **26**, 69.

BALDWIN, E.—(1947) ' Dynamic Aspects of Biochemistry.' London (Cambridge University Press).

BERTALANFFY, L. VON.—(1947) Student (Vienna), **2**, 3.

BERENBLUM, I.—(1929) *Brit. J. Exp. Path.*, **10**, 179.

BOYLAND, E.—(1952) *Cancer Res.*, **12**, 77.

*Idem* AND HORNING, E.—(1949) *Brit. J. Cancer*, **3**, 118.

BUTLER, J. A. V., AND CONWAY, B. E.—(1952) *J. chem. Soc.*, 834.

COOK, J. W., HIEGER, I., KENNAWAY, E. L., AND MAYNEORD, W. V.—(1932) *Proc. Roy. Soc.* B, **111**, 455.

DARLINGTON, C. D., AND KOLLER, P. C.—(1947) *Heredity*, **1**, 187.

DAWSON, J. W.—(1925) ' The Melanomata.' London (Oliver & Boyd).

FOWLER, R. H., AND GUGGENHEIM, E. A.—(1939) ' Statistical Thermodynamics.' London (Cambridge University Press.)

GOPAL-AYENGAR, A. R.—(1953) (in press).

HADDOW, A.—(1938) *Acta Un. int., Cancr.*, **3**, 342.—(1947) *Brit. med. Bull.*, **4**, 331.

*Idem*, HORNING, E., AND TIMMIS, G. M.—(1953) *J. R. micr. Soc.* (in press).

*Idem* AND TIMMIS, G. M.—(1951) *Acta Un. int. Cancr.*, **8**, 469.

HARTWELL, J. L.—(1951) *Publ. Hlth Serv. Publ., Wash.*, No. 149.

HESTON, W. E.—(1950) *J. nat. Cancer Inst.*, **11**, 415.

HINSHELWOOD, C. N.—(1952) *J. chem. Soc.*, 745.

NETTLESHIP, R., AND HENSHAW, P. S.—(1943) *J. nat. Cancer Inst.*, **4**, 309.

OPPENHEIMER, B. S., OPPENHEIMER, E. T., AND STOUT, A. P.—(1948) *Proc. Soc. exp. Biol. N.Y.*, **67**, 33.

PATAT, F.—(1953) *Naturwissenschaften*, **12**, 325.

RASHEVSKY, N.—(1945) *Bull. math. Biophys.*, **7**, 49.

ROBERTS, E., AND TISHKOFF, G. H.—(1949) *Science*, **109**, 229.

RONDONI, P.—(1939) IV Congresso internazionale di patologia comparata, **2**, 52.—(1942) *Ost. ChemZtg.*, **45**, 97.
ROUS, P.—(1936) *Amer. J. Cancer*, **28**, 233.
SCHMIDT, O.—(1939) *Z. phys. Chem.*, **44**, 185.
SCHOENHEIMER, R.—(1949) 'The Dynamic State of Body Constituents.' Cambridge, Mass. (Harvard University Press).
SMITH, E. F.—(1916) *Science*, **43**, 871.
TURNER, F. C.—(1941) *J. nat. Cancer Inst.*, **2**, 81.
TYLER, A.—(1939) 'The Energetics of Embryonic Differentiation, Causal and Chemical Embryology.' Ed. Needham, Paris (Hermann & Cie).
WAUGH, D. F.—(1946) *J. Am. chem. Soc.*, **68**, 247.